# The Relationships between Foot Arch Volumes and Dynamic Plantar Pressure during Midstance of Walking in Preschool Children

**DOI:** 10.1371/journal.pone.0094535

**Published:** 2014-04-15

**Authors:** Hsun-Wen Chang, Hsiao-Feng Chieh, Chien-Ju Lin, Fong-Chin Su, Ming-June Tsai

**Affiliations:** 1 Department of Biomedical Engineering, National Cheng Kung University, Tainan, Taiwan; 2 Department of Physical Therapy, Fooyin University, Kaohsiung, Taiwan; 3 Medical Device Innovation Center, National Cheng Kung University, Tainan, Taiwan; 4 Department of Mechanical Engineering, National Cheng Kung University, Tainan, Taiwan; University of South Australia, Australia

## Abstract

**Objectives:**

The purpose of this study was to examine the correlation between the foot arch volume measured from static positions and the plantar pressure distribution during walking.

**Methods:**

A total of 27 children, two to six years of age, were included in this study. Measurements of static foot posture were obtained, including navicular height and foot arch volume in sitting and standing positions. Plantar pressure, force and contact areas under ten different regions of the foot were obtained during walking.

**Results:**

The foot arch index was correlated (*r* = 0.32) with the pressure difference under the midfoot during the foot flat phase. The navicular heights and foot arch volumes in sitting and standing positions were correlated with the mean forces and pressures under the first (*r* = −0.296∼−0.355) and second metatarsals (*r* = −0.335∼−0.504) and midfoot (*r* = −0.331∼−0.496) during the stance phase of walking. The contact areas under the foot were correlated with the foot arch parameters, except for the area under the midfoot.

**Conclusions:**

The foot arch index measured in a static position could be a functional index to predict the dynamic foot functions when walking. The foot arch is a factor which will influence the pressure distribution under the foot. Children with a lower foot arch demonstrated higher mean pressure and force under the medial forefoot and midfoot, and lower contact areas under the foot, except for the midfoot region. Therefore, children with flatfoot may shift their body weight to a more medial foot position when walking, and could be at a higher risk of soft tissue injury in this area.

## Introduction

The prevalence of flexible flatfoot is 21–57% in children of preschool age [1], [2] and this may lead to further abnormalities and cause pain or adversely influence the performance of physical tasks and walking [2], [3]. According to Lin (2001) [2] and D’Amico (1984) [3], children with flatfoot perform physical tasks poorly, and may develop gait disorders.

The medial longitudinal arch (MLA) plays an important role in allowing the foot to transfer weight and absorb shock when walking or running [4]. According to the height of the MLA, the foot can be classified into three types: high arch, normal arch and flat arch [5]. Previous studies have indicated that MLA type is related to lower extremity injury. Individuals with a high arch may be at an increased risk of injury, including bone injuries [6] (e.g., stress fractures) and lateral injuries (e.g., greater trochanter bursitis, iliotibial band syndrome and/or lateral ankle sprains), while low-arch individuals are at increased risk of soft tissue injuries, medial injuries, and general knee pain [7]. Understanding the correlation between different types of MLA and the risk of injury, and correctly identifying the type of MLA could thus be helpful in predicting musculoskeletal pathology and overuse injury, and providing preventive interventions.

Plantar pressure assessments are widely used to evaluate dynamic foot functions and loading patterns. There are many factors that contribute to the pattern of loading under the foot when walking, such as walking speed, [8] [9] [10] and morphological characteristics [11], [12]. Foot pressure distribution has been related to different types of foot [13], [14], and individuals with flat feet have been found to have decreased peak pressure and maximum force over the lateral forefoot, with increased contact area and maximum force over the medial midfoot [14] and subhallucal area [15].

Dynamic foot motion is commonly measured usinga motion analysis system, which is expensive, and the administration procedure is usually time-consuming. As a result, clinicians commonly use anthropometric or foot print measurements to evaluate the static performance of the medial longitudinal arch to represent the dynamic foot performance during walking and running [16]–[19]. However, it remains unclear to what extent the static appearance of the foot arch can predict dynamic foot arch behavior. Some studies have proposed that static foot arch measurements have a significant correlation with dynamic measurements [16], [17], while others have different results. For example, Trisha et al. [18] proposed that a static measure of the MLA could not predict its dynamic motion. The positive approach, such as that of Cavanagh et al. (1997) [19] has shown that arch related measurements and soft tissue thickness were the strongest predictors of plantar pressure under the heel and the first metatarsal head during walking, but was only able to explain 35% of the variance. Jonely [16] stated that there is a relationship between arch foot posture and dynamic foot pressure, although the strength of this relationship was only poor to fair. Most related studies have examined the relationship between plantar pressure and the two dimensional parameters of MLA (e.g., arch height, arch height ratio, navicular drop, navicular drift, and navicular height) [16], [17]. MLA is a multi-site three-dimensional structure which is constructed by the talus, calcaneus, navicular, the three cuneiforms, and the first, second, and third metatarsals. A 2D assessment is thus not able to supply comprehensive information about the MLA, and it is believed that 3D data would provide the most accurate evaluation of the foot arch [20], [21]. However, no studies have been carried out from a three dimensional perspective. The first part of our research used foot arch volume to describe the characteristic of MLA, and showed that it was highly correlated to navicular height and was discriminative from the ages two to six [22]. Therefore, the aim of the second part of our research was to examine the correlation between the foot arch volume measured from static positions and the plantar pressure distribution during walking.

## Methods

### Ethics Statement

This study was approved by the Institutional Review Board of Fooyin University Hospital (FYH-IRB-099-06-03), and written informed consent was obtained from the parents of all the children prior to testing.

### Design

A single group exploratory design using a correlation method was used to determine the correlation between the static functional index and plantar pressure during walking.

### Subjects

Children aged from two to six were enrolled in this study. Subjects were excluded from the study if the following conditions were presented: (i) diagnosis of fixed foot deformity, (ii) pain in the ankle or foot within the last three months, or (iii) evidence of developmental disabilities that may influence the development of the foot. The subjects who were included were part of the sample from the first part of our research which was published in 2012 [22].

### Procedures

#### Clinical anthropometric measurements

The height and weight was measured by a stadiometer and an electronic scale. The foot parameters were measured in a one leg standing position. The width and length of both feet were measured with a digital caliper (Jingstone Precision Measuring & Calibration co., Ltd., Taiwan) with a resolution of 0.01 mm. The foot width was measured between the first and fifth metatarsal heads. The foot length was measured from the most posterior point of the calcaneus to the end of the longest toe. The navicular height (NH) was obtained from the lowest palpable medial projection of the navicular to the supporting surface using a standard steel ruler. The measurements of the foot parameters were all administered in the one leg standing position. All of the measurements were taken by the same experienced physical therapist.

#### Measurement of the foot arch volume

Each foot of the subjects was scanned three times in sitting and standing positions by the “Peripher 3D Scanner” which was designed by the Robotic and Automation Research Laboratory at National Cheng Kung University in Taiwan. When sitting, the subjects sat on a height adjustable chair and were asked to keep the hip and knee joints at 90° of flexion and the ankle joint in a neutral position. When standing, the subjects stood on one leg and kept all the following landmarks in alignment, including the acromion process, hip center, knee joint center and lateral malleolus. One foot was scanned at a time, and the unscanned leg was suspended in front on a suspended board.

#### Plantar pressure distribution of the foot during gait

Plantar pressure measurements were obtained using a Footscan 3D system with Footscan software 7.1 (RSscan International, Olen, Belgium). This system consists of an eight mm thick pressure platform (500 mm×400 mm in length and width) incorporating 4,096 sensors (5.3×7.5 mm/sensor) with 0.27–127 N/sqcm sensitivity and a sampling rate of 500 Hz. The pressure platform was positioned at the center of a 15-meter long and two-meter wide walkway. The pressure platform was calibrated for each subject using their own bodyweight prior to each testing session.

Following verbal instruction and demonstration, all subjects were directed to walk along the walkway barefooted at a self-selected speed. Before data collection, the subject was instructed to practice three to five trials to be familiar with these testing movements. Once the subject was comfortable with the task, data collection was started and continued for ten successful trials, with five recordings for each foot. A successful trial was defined as only one foot stepping on the pressure plate and the subject demonstrating a normal gait pattern. The data from an average of the 2^nd^ to 4^th^ trials on the pressure plates were used to represent the individual’s dynamic pressure pattern.

#### Data processing

The 3D foot models were constructed using the Geomagic Studio 10 (N.C., U.S.A.) commercial software package. The foot arch volume was calculated based on the arch plane projecting to the supporting plane. The arch volume index (AVI) was calculated based on the difference in the foot arch volume in sitting and standing conditions, using the following method presented by Chang (2012) [22].
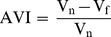



In this, V_n_ and V_f_ represent the volume under the foot arch in a non-weight bearing (sitting) and full weight bearing (one leg standing) position, respectively.

**Figure 1:**
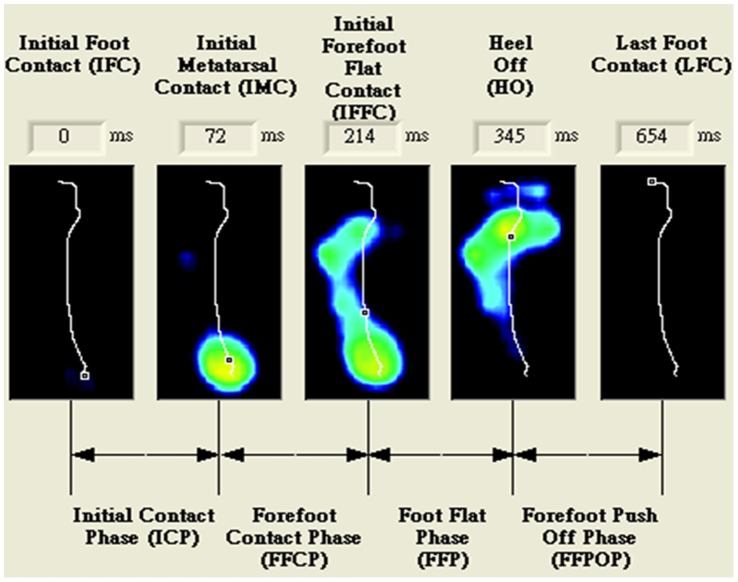
The representative figure for definition of the four subphases of the stance phase in walking.

The foot pressure distribution was processed using FootScan software 7.0. The foot was divided into ten regions: big toe, 2^nd^–5^th^ toes, 1^st^ metatarsal, 2^nd^ metatarsal, 3^rd^ metatarsal, 4^th^ metatarsal, 5^th^ metatarsal, midfoot, lateral heel and medial heel. We grouped the big toe, 2^nd^–5^th^ toes, 1^st^ metatarsal, 2^nd^ metatarsal, 3^rd^ metatarsal, 4^th^ metatarsal and 5^th^ metatarsal as the forefoot, and the lateral and medial heels as the hindfoot. The contact area, force and pressure values were assessed for each of the ten foot regions during the four subphases of the stance gait cycle. The four phases were the initial contact phase (phase I), the forefoot contact phase (phase II), the foot flat phase (phase III) and the forefoot push off phase (phase IV), which were defined by the following specific timings: initial foot contact, initial metatarsal contact, initial forefoot flat contact, heel off and last foot contact, respectively, as shown in Figure 1.

The foot flat phase starts from the full forefoot contact and ends with the heel off, which represents that the body weight is supported from the double supporting phase to the single stance phase. The foot arch is loaded from partial weight bearing to full weight bearing. The change in pressure during the foot flat phase is used to represent the dynamic performance of the foot.

### Statistical Analysis

Statistical analysis was performed using the SPSS 17 software. The relationships between the foot arch indices and mean plantar pressure, force and contact areas on different regions under the foot during walking were explored using Pearson correlation coefficients. A significance level of a *p*-value less than 0.05 was used for all the analyses.

## Results

A total of 27 children (nine boys and 18 girls) were enrolled in this study. The mean age was 55 months, and ranged from 25 to 75 months. The demographic data are presented in Table 1.

**Table 1:** Subject characteristics and descriptive results of static foot arch variables, with the values presented as means (SD) and ranges.

N = 54 (feet)	Mean (SD) [min = max]
**Age** (months)	55 (17.08) [25–75]
**Height** (cm)	104 (11.30) [81–119]
**Weight** (kg)	17 (4.18) [9]–[27]
**Body mass index** (kg/m^2^)	15.85 (2.32) [13]–[25]
**AVI**	0.47 (0.127) [0.10–0.47]
**V_f_** (mm^3^)	5352.81 (2380.305)[1421.30–13040.00]
**V_n_** (mm^3^)	10007.96 (3647.148) [3726.20–21147.50]
**NH** (mm)	20.90 (4.51) [11.00–29.00]

N: number; SD: standard deviation; min: minimal value; max: maximal value; AVI: arch volume index; V_f_: arch volume in standing position; V_n_: arch volume in sitting position; NH: navicular height.

The mean ± SD values for the static foot arch parameters, including AVI, navicular height, and foot arch volume in sitting and standing conditions, are also listed in Table 1. The mean forces under the forefoot, midfoot, and hindfoot during the entire stance phase were 119.09, 35.59, 58.45 N, respectively, and the mean plantar pressures under the forefoot, midfoot, and hindfoot during the stance phase were 2.99, 3.70, 3.94 N/cm^2^, respectively. The mean forces under the 10 foot regions during all four subphases are shown in Table 2.

**Table 2:** The mean forces (N) and standard deviations under different foot regions during four phases (N = 54 feet).

	Toes		Forefoot					Midfoot	Hindfoot		Sum
	Toe1	Toe2–5	Meta1	Meta2	Meta3	Meta4	Meta5	Midfoot	Heel Med	Heel Lat	
**Phase I**	.00(.00)	.00(.00)	.02(.14)	.03(.019)	.04(.025)	.18(.1,28)	1.05(4.62)	22.00(23.30)	71.86(44.89)	62.17(39.91)	157.72(90.10)
**Phase II**	.79(2.87)	.12(.71)	2.23(5.25)	3.19(6.68)	2.19(4.26)	2.18(5.23)	1.57(4.83)	56.78(47.06)	89.11(44.76)	61.98(34.67)	219.66(98.45)
**Phase III**	3.89(12.84)	.25(.60)	15.44(20.42)	27.29(23.13)	21.68(21.10)	18.73(13.58)	7.00(7.90)	95.36(66.49)	40.56(27.26)	24.63(20.51)	254.82(110.67)
**Phase IV**	37.87(35.11)	7.50(8.11)	22.27(22.21)	42.69(23.84)	44.68(29.07)	26.02(18.03)	8.39(9.45)	7.42(11.47)	0.19(1.22)	0.26(1.37)	197.66(90.02)
**Stance phase**	21.01(20.43)	3.79(4.31)	14.26(13.47)	27.91(15.49)	28.03(21.11)	17.91(12.42)	6.18(7.08)	35.59(29.43)	33.87(18.78)	24.58(16.81)	213.03(91.14)

Meta1: the first metatarsal area; Meta2: the second metatarsal area; Meta3: the third metatarsal area; Meta4: the fourth metatarsal area; Meta5: the fifth metatarsal area; Heel Med: the medial area of heel; Heel Lat: the lateral area of heel.

The Pearson product moment correlation coefficients for the foot arch parameters in the static position in relation to the change in dynamic foot pressures measured under the three regions of the foot during the foot flat phase are shown in Table 3. The interpretation of the Pearson’s coefficient *r* is according to Portney [23] and Milton [24] who state that a correlation ranging from 0.25 to 0.50 suggests a fair degree of relationship, a value of 0.5 to 0.75 is moderate to good, while a value above 0.75 is considered good to excellent. AVI has a fair correlation (*r = *0.320) with the change in plantar pressure under the midfoot (*p*<0.05), while V_f_ has a weak correlation (*r = *0.286) with the change in plantar pressure under the hindfoot (*p*<0.05). Except for these two indexes, no other correlations were found with pressure difference under the foot.

**Table 3:** The correlation between foot arch parameters and the change in foot pressure during the foot flat phase.

	ΔForefoot P	ΔMidfoot P	ΔHindfoot P	ΔEntire foot P
AVI	−0.106 (0.810)	0.320*(0.022)	−0.087 (0.544)	0.261(0.064)
V_f_	−0.202 (0.159)	−0.200 (0.874)	0.286*(0.042)	0.027(0.390)
V_n_	−0.213 (0.155)	−0.079 (0.159)	0.258 (0.067)	−0.078(0.850)
NH	−0.200 (0.134)	−0.023 (0.582)	0.170 (0.233)	−0.123(0.587)

* *p*<0.05.

AVI: arch volume index; V_f_: arch volume in standing position; V_n_: arch volume in sitting position; NH: navicular height; P: pressure; Δ: the difference.

The correlations between the mean forces, pressures and contact areas under the 10 foot areas and the foot arch parameters are listed in Table 4. The mean forces under Meta1, Meta2, Midfoot and the entire foot were significantly fairly correlated with V_f_ (*r* = −0.355∼−0.481) and V_n_ (*r* = −0.329∼−0.504). NH was only negatively correlated with the mean forces under Meta1 and Meta2. The results for the mean pressures were similar to those for the mean forces. The contact areas under most of the foot regions were correlated with NH, V_f_ and V_n_. AVI was not correlated with the mean forces, pressures and contact areas under the different regions of the foot during the entire stance phase except for the pressure under Heel Lat. Although there were some correlations between the static arch parameters and plantar pressures, the strengths of these relationships were only fair. A higher correlation was only shown between V_n_ and V_f_ and the contact areas under Heel Lat. and Heel Med.

**Table 4:** The correlation between arch parameters and force, pressure and contact area under ten foot regions during the stance phase.

		AVI	NH	V_f_	V_n_
**Toe1**	force	−0.049	−0.147	−0.219	−0.288*
	pressure	0.007	−0.153	−0.218	−0.255
	contact area	−0.276	0.447**	0.529**	0.462**
**Toe2–5**	force	0.150	0.149	−0.192	−0.167
	pressure	0.117	0.104	−0.171	−0.145
	contact area	−0.037	0.456**	0.450**	0.506**
**Meta1**	force	0.199	−0.312*	−0.355*	−0.329*
	pressure	0.156	−0.296*	−0.355*	−0.346*
	contact area	−0.032	0.407**	0.558**	0.604**
**Meta2**	force	0.083	−0.373**	−0.455**	−0.504**
	pressure	0.092	−0.335*	−0.418**	−0.463**
	contact area	−0.084	0.490**	0.539**	0.597**
**Meta3**	force	−0.108	−0.193	−0.162	−0.215
	pressure	−0.062	−0.122	−0.114	−0.152
	contact area	−0.187	0.371**	0.503**	0.522**
**Meta4**	force	−0.178	−0.012	−0.121	−0.207
	pressure	−0.152	−0.002	−0.069	−0.137
	contact area	−0.161	0.475**	0.494**	0.486**
**Meta5**	force	−0.085	0.258	0.083	0.056
	pressure	−0.147	0.302*	0.187	0.139
	contact area	0.235	0.384**	0.120	0.286**
**Midfoot**	force	0.202	−0.164	−0.481**	−0.496**
	pressure	0.129	−0.043	−0.331*	−0.360*
	contact area	0.125	0.105	−0.123	−0.061
**Heel Lat.**	force	0.110	−0.107	0.034	0.100
	pressure	0.321*	−0.066	−0.100	0.054
	contact area	−0.169	0.467**	0.735**	0.729**
**Heel Med.**	force	0.271	−0.135	−0.132	−0.008
	pressure	0.146	−0.053	0.071	0.158
	contact area	−0.217	0.464**	0.713**	0.729**
**Entire foot**	force	0.122	−0.261	−0.393**	−0.404**
	pressure	0.094	−0.157	−0.254	−0.260
	contact area	−0.053	0.531**	0.524**	0.587**

**Correlation is significant at the 0.01 level.

*Correlation is significant at the 0.05 level.

Meta1: the first metatarsal area; Meta2: the second metatarsal area; Meta3: the third metatarsal area; Meta4: the fourth metatarsal area; Meta5: the fifth metatarsal area; Heel Med: the medial area of heel; Heel Lat: the lateral area of heel; AVI: arch volume index; V_f_: arch volume in standing position; V_n_: arch volume in sitting position; NH: navicular height.

## Discussion

Chang (2012) used the foot arch volume to investigate MLA [22]. The foot arch volume parameters were measured by a three dimensional laser scanner, and the results showed a moderate correlation with navicular height, and could thus be used to discriminate arch development with age. In addition, according to Chang (2012), the foot arch volumes were significantly different in children from two to six years old, and thus can be used to discriminate the stages of arch development at various ages. The issue of the prediction of dynamic foot function from static foot arch measurements remains controversial. In contrast, AVI is a new index which is able to represent foot arch flexibility and indicate the change in mechanical energy due to the foot arch deformations. The aim of this study was thus to explore the relationships among AVI, V_n,_ and V_f_, and foot plantar pressure values under the forefoot, midfoot, and hindfoot during the flat foot phase while walking.

AVI and V_f_ showed significantly fair correlations with the plantar pressure differences under the foot during the flat foot phase, which suggests that there may be a meaningful relationship between foot arch characteristics and pressure distribution during walking. AVI demonstrated a fair correlation with plantar pressure difference under the midfoot, which suggests that the flexibility of the MLA will influence the dynamic change in pressure distribution under the foot during midstance. Subjects with a more flexible MLA would tend to produce a higher change in plantar pressure under the midfoot for the time between forefoot contact to heel off. The flat foot phase is defined in this study as from forefoot contact to heel off, when the body weight shifts from the opposite limb to the loading limb. The MLA undergoes deformation due to increasing body weight in this phase, and this changes the pressure distribution. We assume that different flexibilities with regard to the MLA will cause this pressure distribution to differ. The results of this study show that AVI was correlated with plantar pressure under the midfoot, although the strength of this correlation was not very strong. V_f_ is correlated with the change in plantar pressure under the hindfoot. The results show that subjects with a higher arch tended to have a greater change in plantar pressure under the hindfoot during the midstance phase. In summary, the strength of these correlations was not very strong, which may indicate that the arch flexibility and arch height represented by AVI and V_f_ are just two of the factors which will influence the change in plantar pressure under the foot during walking. There are still other unmeasured factors such as plantar tissue thickness and muscle action [25] which should be focused on.

The results of this study show that there was correlation between foot arch parameters and the mean pressures and forces under the midfoot, first and second metatarsal regions; however, the strength of this correlation may be only poor to fair. The association between a declining V_n_ and V_f_ and increased midfoot loading is consistent with Morag and Cavanagh [25] and Menz et al. [26], who found that the arch index was significantly associated with peak midfoot pressure. A declining V_n_ and V_f_, which is representative of a flatter foot, demonstrated a significant medial longitudinal arch lowering during the midstance phase of gait. The lowering movement pattern apparently increases loading under the midfoot. The results related to the forces and pressures under the medial forefoot are consistent with Ledoux et al. (2002) [15], who found that subjects with flatfoot had significantly more force in the subhallucal and first metatarsal area. Chuckpaiwong et al. [14] compared the peak pressure and maximum force between normal and low arch subjects, and concluded that the latter showed significant decreases in load in the lateral forefoot, which suggests that low arch subjects will shift their bodyweight to the medial forefoot. Teyhen et al. (2009) [27] also concluded that there was a positive association between a higher arched foot and increased pressure in the lateral forefoot. In this study, we found similar results in that the lower MLA affected the medial weight shift more obviously on the forefoot, not on the rearfoot. In contrast, Jonely et al. [16] found a lower peak pressure under the medial forefoot for low arch subjects. The differences between these results could be due to many reasons, including the different arch related measurements, the different foot area definitions, and the different ages of the subjects. Children were the subjects in the current study, while other works recruited adults or elderly subjects, and the pressure distribution is known to differ between children and adults, especially for younger children [28]. Jonely used arch index and navicular drop to represent foot arch, and only focused on investigating the peak pressure over the medial side of the foot; however, the volume under the foot was used to represent the foot arch in this study, and we investigated the pressure distribution under the entire foot. These factors mean that the results are difficult to compare.

The most significant result obtained in this study was that the static foot arch parameters were significantly (*p*<0.05) correlated with the contact areas under the entire foot (*r* = 0.524∼0.587), especially for Heel Lat (*r* = 0.467∼0.735) and Heel Med (*r* = 0.464∼0.729). The foot arch parameters were positively correlated with the contact area under the foot, which indicated that those individuals with a lower arch will demonstrate a smaller contact area under the heel. This may be due to the weight distribution from other regions of the foot to the midfoot on the subjects with a lower arch. Decreasing weight bearing will decrease the contact area [29]. According to the literature, the contact area was smaller under the midfoot in subjects with pes cavus [6], [14]. However, we did not conclude the same result over the midfoot.

Most previous studies examined the relationships between MLA measurements and regional peak plantar pressure values during the entire stance phase of walking, with no focus on the midstance phase. In this study, we investigated the change in pressure during the midstance phase to explore the function of MLA at this time. The results will help us understand how MLA affects weight distribution during the midstance phase of walking.

## Conclusion

AVI was significantly related to the change in pressure under the midfoot during the flat foot phase of walking. This implies that the AVI measured in a static position is able to correlate with dynamic changes in foot loading under the midfoot. However, it is not able to correlate with the mean pressure and force during the entire stance phase. The NH, V_f_ and V_n_ were significantly related to the mean pressure and forces under the 1^st^ and 2^nd^ metatarsals and the midfoot throughout the entire stance phase. As the arch height decreases, the medial metatarsals and midfoot plantar pressures and forces increase. The foot arch volumes had a higher correlation with the pressure distribution under the foot compared to NH, which suggests that foot arch volume may be a more sensitive way to detect the foot arch than NH. These results reveal some important patterns of plantar pressure distribution in feet with different foot arch heights, and indicate that lower arch subjects may easily develop ulceration under the medial side of the foot.
